# Accumulation of Ambient Black Carbon Particles Within Key Memory-Related Brain Regions

**DOI:** 10.1001/jamanetworkopen.2024.5678

**Published:** 2024-04-09

**Authors:** Kenneth Vanbrabant, Debby Van Dam, Eva Bongaerts, Yannick Vermeiren, Hannelore Bové, Niels Hellings, Marcel Ameloot, Michelle Plusquin, Peter Paul De Deyn, Tim S. Nawrot

**Affiliations:** 1 Centre for Environmental Sciences, Hasselt University, Diepenbeek, Belgium; 2 Laboratory of Neurochemistry and Behaviour, Experimental Neurobiology unit, Department of Biomedical Sciences, University of Antwerp, Antwerp, Belgium; 3 Division of Human Nutrition and Health, Chair Group of Nutritional Biology, Wageningen University & Research (WUR), Wageningen, the Netherlands; 4 Hasselt University, Department of Sciences, Diepenbeek, Belgium; 5 Biomedical Research Institute, Hasselt University, Diepenbeek, Belgium; 6 Hasselt University, Biophysics, Diepenbeek, Belgium; 7 Department of Neurology and Alzheimer Research Center, University of Groningen and University Medical Center Groningen, Groningen, the Netherlands; 8 Faculty of Medicine & Health Sciences, Translational Neurosciences, University of Antwerp, Antwerp, Belgium

## Abstract

**Importance:**

Ambient air pollution is a worldwide problem, not only related to respiratory and cardiovascular diseases but also to neurodegenerative disorders. Different pathways on how air pollutants could affect the brain are already known, but direct evidence of the presence of ambient particles (or nanoparticles) in the human adult brain is limited.

**Objective:**

To examine whether ambient black carbon particles can translocate to the brain and observe their biodistribution within the different brain regions.

**Design, Setting, and Participants:**

In this case series a label-free and biocompatible detection technique of nonincandescence-related white light generation was used to screen different regions of biobanked brains of 4 individuals from Belgium with neuropathologically confirmed Alzheimer disease for the presence of black carbon particles. The selected biological specimens were acquired and subsequently stored in a biorepository between April 2013 and April 2017. Black carbon measurements and data analysis were conducted between June 2020 and December 2022.

**Main Outcomes and Measures:**

The black carbon load was measured in various human brain regions. A Kruskal-Wallis test was used to compare black carbon loads across these regions, followed by Dunn multiple comparison tests.

**Results:**

Black carbon particles were directly visualized in the human brain of 4 individuals (3 women [75%]; mean [SD] age, 86 [13] years). Screening of the postmortem brain regions showed a significantly higher median (IQR) number of black carbon particles present in the thalamus (433.6 [289.5-540.2] particles per mm^3^), the prefrontal cortex including the olfactory bulb (420.8 [306.6-486.8] particles per mm^3^), and the hippocampus (364.7 [342.0-448.7] particles per mm^3^) compared with the cingulate cortex (192.3 [164.2-277.5] particles per mm^3^), amygdala (217.5 [147.3-244.5] particles per mm^3^), and the superior temporal gyrus (204.9 [167.9-236.8] particles per mm^3^).

**Conclusions and Relevance:**

This case series provides evidence that ambient air pollution particles are able to translocate to the human brain and accumulate in multiple brain regions involved in cognitive functioning. This phenomenon may contribute to the onset and development of neurodegenerative disorders.

## Key Points

**Question:**

Do inhaled ambient black carbon particles accumulate in the human brain?

**Findings:**

This case series found evidence of black carbon particles in brain tissue, suggesting translocation and biodistribution, with increased accumulation of particles in key memory-related brain regions such as the thalamus and hippocampus.

**Meaning:**

These results suggest that certain brain regions are more susceptible to the accumulation of air pollutants and may be a first step into elucidating the potential link between air pollution exposure and observed neurological outcomes.

## Teaser

This case series of brain tissue samples from 4 Belgian individuals with confirmed Alzheimer disease assesses the presence of black carbon particles in several brain regions associated with cognitive functioning.

## Introduction

Air pollution is a global concern with both short-term and long-term effects on human health.^1^ Exposure to air pollution, specifically fine particulate matter (PM_2.5_) and combustion-derived particulate matter that includes black carbon, has been linked to a range of health hazards, such as respiratory diseases, cardiovascular diseases, and cancer. In recent years, research has also elucidated that air pollution exposure can have detrimental effects on brain health.^2^ Exposure to air pollution has been associated with cognitive decline, intellectual disability, and increased risk of neurological disorders such as stroke, Alzheimer disease, and Parkinson disease.^2,3^

Air pollution can affect the brain through several mechanisms, such as through the inhalation of black carbon particles. These tiny particles are part of PM_2.5_ and are small enough to enter the bloodstream and travel to the brain.^4,5^ The translocation of black carbon particles to the brain is a complex process that might involve various mechanisms, such as induction of a leaky blood-brain barrier, which serves to protect the brain from harmful substances.^6^ Alternatively, it is hypothesized that air pollutant particles can also enter the brain by traveling across the olfactory nerve, which carries information about the sense of smell from the nose to the brain.^7^ Once in the brain, these particles can trigger an immune response that leads to inflammation and oxidative stress, which in turn can cause damage to neurons and may lead to neurodegeneration.^8,9^

Previously, we found that ambient black carbon particles can cross the human placenta into the fetal circulation and fetal brain.^10^ The presence of black carbon in the fetal brain might have an important impact on brain development, as well as brain health during later life. However, little is known about the continuous accumulation of black carbon particles in the brain throughout life and the biodistribution of these particles within the brain itself. We therefore aimed to determine whether ambient black carbon particles can translocate and accumulate in the adult human brain during the lifespan. Examination of the presence of black carbon particles in different brain regions—including the thalamus, orbitofrontal cortex along with the olfactory bulb, hippocampus, cingulate cortex, amygdala, and gyrus temporalis superior—was performed to gain more insight into the distribution throughout the brain.

## Methods

### Samples

To study the translocation of ambient black carbon particles toward the adult human brain in this exploratory case series, we determined the brain black carbon load in postmortem brain tissue of 4 individuals at high age. Six selected brain regions per individual were screened for the presence of black carbon particles. We used a subset of different paraffin-embedded postmortem brain regions (ie, thalamus at the level of the nucleus centralis, orbitofrontal cortex [Brodmann area 11] along with the olfactory bulb, hippocampus, cingulate cortex [Brodmann area 24], amygdala, and gyrus temporalis superior [Brodmann area 22]) from 4 individuals obtained in collaboration with the University of Antwerp and related biobank between April 2013 and April 2017. The biobank of Antwerp is a repository of biological samples collected from individuals who have consented to store residual samples for research purposes. Using a microtome (Leica Microsystems), 8-μm sections were cut, floated onto charged glass slides (Fisher Scientific), dried overnight at 37° C, and stored at room temperature until analysis. The sample collection and sample use were approved by the ethical committees of University of Antwerp and conducted in compliance with the principles of the Declaration of Helsinki.^11^ This study was performed according to the reporting guideline for case series.

### Procedure for Black Carbon Detection in Brain Tissue

Black carbon particles in the different brain regions were detected via a specific and label-free technique based on the nonincandescence-related white light generation of the particles under femtosecond-pulsed illumination, as previously described by our research group.^10,12,13^ Images were taken with a confocal microscope (Carl Zeiss) equipped with a 2-photon femtosecond-pulsed laser (810 nm, 150 fs, 80 MHz) (Spectra-Physics) using a Plan-Neofluar 10x/0.3 objective (Carl Zeiss). Two-photon–induced white light emission of carbon particles was acquired in the nondescanned mode after spectral separation and emission filtering using 400-410 nm and 450-650 nm band-pass filters. The resulting tile scans of 13.35 μm × 13.35 μm were recorded with a 1.66 μm pixel size and 4.10 μs pixel dwell time.

### Validation Experiments of White Light From Black Carbon Brain Tissue

As previously described by our research group,^10^ we validated the carbonaceous nature of the detected black carbon particles based on 2 of the characteristic white light features: (1) emitted white light ranges over the whole visible spectrum, and (2) the temporal response of black carbon particles is known to be instantaneous. Additional optical sectioning in the Z-direction throughout the brain tissue was performed to show tissue embedment of black carbon particles. Approximately 100 images of each 512 by 512 pixels and with voxels of 1.137 × 1.137 × 0.500 μm^3^ were acquired throughout the tissue sections using a pixel dwell time of 4.10 μs. Orthogonal XZ- and YZ-projections were made using the software Fiji. All validation experiments were performed using a confocal laser scanning microscope with a Plan-Aprochromat 20x/0.8 objective (Carl Zeiss) suitable for nonlinear optical imaging.

### Statistical Analysis

The analysis of the black carbon load within the 5 observed brain regions was carried out using commercially available Prism version 8 (GraphPad Software Inc) and presented as median (IQR). Study population characteristics are expressed as means (SDs) or numbers. Normality was tested using the Shapiro-Wilk test. Despite conforming to a normal distribution, a Kruskal-Wallis test was employed to compare medians across the regions due to the small sample size. Statistically significant differences in the black carbon load of the thalamus, orbitofrontal cortex, hippocampus, cingulate cortex, amygdala, and the superior temporal gyrus were analyzed using the Kruskal-Wallis test with unadjusted Dunn multiple comparisons. We considered *P* < .05 in 2-sided tests to be statistically significant.

## Results

Our study included postmortem samples from patients with neuropathologically confirmed Alzheimer disease (3 women, 1 man; mean (SD) age, 86 (13) years) who were not active smokers at the time of death and who had lived in an urban environment in the Flemish-speaking part of Belgium. Neuropathological diagnosis was performed according to the updated consensus National Institute on Aging–Alzheimer Association guidelines using the ABC score derived from 3 separate 4-point scales. These scales include quantification of amyloid-β plaque by the Thal phases method (expressed as *A* and ranging from A0 [indicating no Aβ or amyloid plaques] to A3 [Thal phase 4 or 5]), neurofibrillary tangle (NFT) by the Braak method (B, ranging from B0 [no NFTs] to B3 [Braak stage V or VI]), and neuritic plaque load by the Consortium to Establish a Registry for Alzheimer disease (CERAD) method (C, ranging from C0 [no neuritic plaques] to C3 [indicating frequent neuritic plaques]).^14^ Using this ABC score system, 1 individual had a composite score of A3B3C2, 2 individuals had a composite score of A3B3C2-3, and 1 individual had a composite score of A3B3C3. These scores correspond to fully developed Alzheimer disease pathology according to Montine criteria.^15^

**Figure 1.**
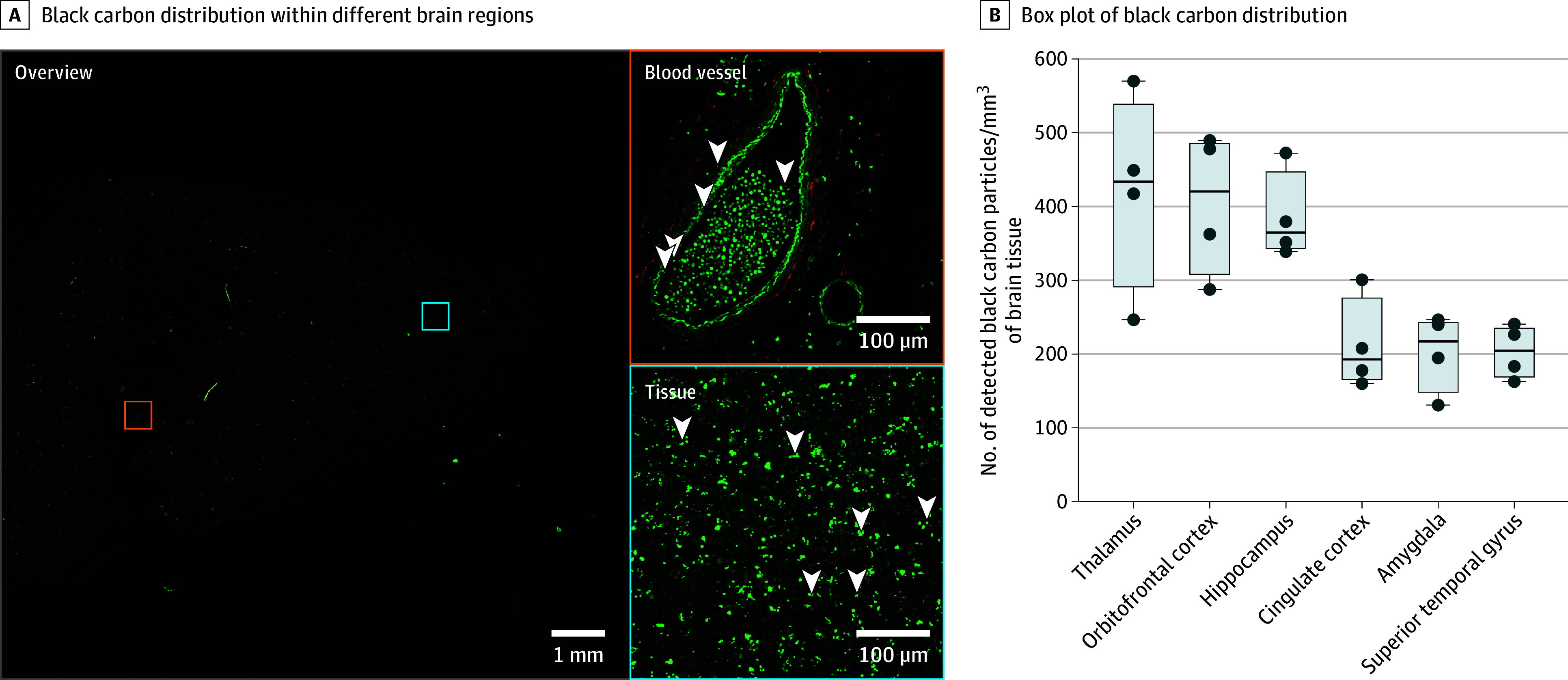
Black Carbon Distribution Within Different Brain Regions of Postmortem Brain Tissue From Patients With Alzheimer Disease A, The presence of intra-tissue black carbon particles within human brain tissue (blue square) and around the blood vessels (orange square) of the hippocampus, depicted in white and indicated with white arrowheads in the zoomed-in squares on the right. B, Boxplots illustrate measured black carbon particle load in 6 different brain regions. Carbonaceous particles were detected via nonincandescence related white light generation under femtosecond-pulsed illumination. The black line inside the boxes indicates the median; the edges of the boxes, first and third quartiles; the whiskers, the largest and smallest value; and the blue dots, the individual particle load for the different brain regions (4 regions).

Analysis of black carbon in the brain revealed that particles could not only be observed in and around the cerebral blood vessels, but could also be detected dispersed throughout the brain tissue itself (Figure 1A). Black carbon particles were present in all the analyzed brain regions (Figure 1B). Compared with the cingulate cortex (192.3 [164.2-277.5] particles per mm^3^), the amygdala (217.5 [147.3-244.5] particles per mm^3^), and the gyrus temporalis superior (204.9 [167.9-236.8] particles per mm^3^), the median black carbon load of the thalamus (433.6 [289.5-540.2] particles per mm^3^), the orbitofrontal cortex along with the olfactory bulb (420.8 [306.6-486.8] particles per mm^3^), and the hippocampus (364.7 [342.0-448.7] particles per mm^3^) was significantly higher with an approximately 2-fold increase (Table). The highest number of particles was detected in the thalamus, followed by the orbitofrontal cortex and the hippocampus, respectively. Moreover, the cingulate cortex, the amygdala, and the gyrus temporalis superior had an almost equal number of particles present.

**Table.** *P* Value Results of the Multiple Comparison of Black Carbon Load in the Different Examined Brain Regions^a^

	Thalamus	Orbitofrontal cortex	Hippocampus	Cingulate cortex	Amygdala	Gyrus temporalis superior
Thalamus	NA	NA	NA	NA	NA	NA
Orbitofrontal cortex	>.99^b^	NA	NA	NA	NA	NA
Hippocampus	.80^b^	.80^b^	NA	NA	NA	NA
Cingulate cortex	.02	.02	.03	NA	NA	NA
Amygdala	.02	.02	.03	>.99^b^	NA	NA
Gyrus temporalis superior	.01	.01	.03	.96^b^	.96^b^	NA

Abbreviation: NA, not applicable.

^a^ The Kruskal-Wallis test was employed for all data followed by Dunn multiple comparison test (α = .05).

^b^ Nonsignificant.

**Figure 2.**
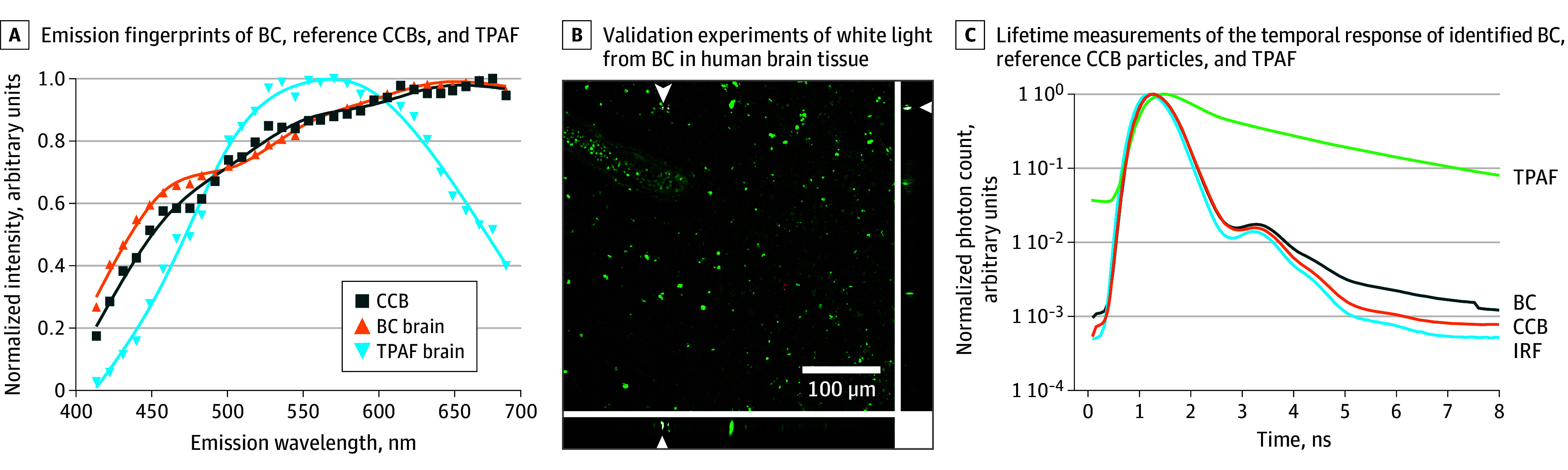
Validation Experiments of White Light From Black Carbon (BC) in Human Brain Tissue A, Emission fingerprints of BC, reference conductive carbon black particles (CCB), and 2-photon autofluorescence (TPAF) were measured for the brain tissue under femtosecond-pulsed illumination. B, Image in XY-projection acquired throughout sectioning of brain tissue in the z-direction, showing a BC particle (white, indicated by white arrowheads) in the corresponding orthogonal XZ-projection and YZ-projection inside the tissue (green, autofluorescence; red, second harmonic generation). C, Lifetime measurements show the temporal response of identified BC, reference CCB particles, and TPAF. IRF indicates instrument response function.

To confirm the carbonaceous nature of the particles, rigorous validation experiments were performed. The characteristic fingerprint of the emitted white light generated by the black carbon particles inside the brain tissue shows a signal spread over a wide range of emission wavelengths comparable with the signal of commercially engineered carbon black (CCB) (Figure 2A).^12^ This contrasts with the emission fingerprint of autofluorescence signals of the brain tissue consisting of a distinct peak that does not range over all wavelengths. Optical sectioning in the Z-direction (ie, along the axis perpendicular to the optical sectioning of the brain slice) throughout the brain tissue and the corresponding orthogonal projections show the embedment of particles inside the brain tissue and were, therefore, not originating from external contamination during sample preparation (Figure 2B). Finally, we observed that the recorded temporal response of the black carbon and the reference particle were nonresolvable from the instrument response function (indicating an instantaneous response) while the temporal response recorded from the autofluorescent signal of the brain tissue showed an exponential decay (Figure 2C).

## Discussion

It is known that air pollutants can reach the brain and exert ample deleterious effects leading to neurodegenerative diseases and central nervous system impairments.^4,5,16^ The presence of ambient black carbon particles in the aged human brain was studied in depth using samples from 6 different brain regions. We not only observed the presence of black carbon particles within brain tissue, which provides evidence of their transfer to the adult human brain, but we also demonstrated increased accumulation in key memory-related brain regions.

The significantly elevated number of particles in the orbitofrontal cortex, including the olfactory bulb, suggests that a substantial part of environmental black carbon particles can enter the adult human brain via the olfactory pathway,^7,17^ after which it accumulates in the aforementioned regions. However, the observed increase in particle load in brain regions like the thalamus or the hippocampus does not exclude the possibility of multiple entry routes. Within the different brain regions, we could observe black carbon particles around and in close proximity to some of the blood vessels. These residual detected particles in the other observed regions were expected to originate from the direct pathway of translocation from the lungs to the brain over the blood-brain barrier.^18^ Hence, black carbon particles were expected to be equally distributed over the remainder of the brain. Contradicting that expectation, significantly increased numbers of particles, similar to the number of particles in the olfactory bulb, could also be observed in regions located more deeply of the brain, ie, the hippocampus in the mesencephalon and the thalamus in the diencephalon. The hippocampus is part of the limbic system and plays a major role in short- and long-term declarative learning and memory, as well as in spatial memory. Experimental studies on carbon in rats and titanium dioxide nanoparticles in mice demonstrate that the highest number of particles are in the olfactory bulb and hippocampus with lower levels in the cortex after whole body or intranasal exposure, respectively, confirming our results.^19,20^ The thalamus is mainly known as a relay station to cortex of multiple inputs, including pathways related to motor function, limbic function, attention, and sleep from other structures in the brain or in the periphery. Additionally, the thalamus is also established to play an important role in the formation and storage of memories. The thalamus acts as a crossroads within the brain, connecting distant brain regions.^21^ These connections may play a role in the translocation of black carbon particles within the brain, as it is hypothesized that air pollutant nanoparticles can travel along the axonal path of the brain.^7^ However, we can only speculate about the intratissue translocation of black carbon in the brain with the available data. Biokinetic studies are needed to investigate further the distribution of these black carbon particles in the human brain.

All other brain regions (cingulate cortex, amygdala, and gyrus temporalis superior) show relatively low but still augmented amounts of particle accumulation. The cingulate cortex plays a key role in emotional regulation and cognitive decision-making, and the gyrus temporalis superior is involved in the perception of emotional stimuli. Interestingly, both long-term^22,23^ and recent^22^ exposure has already been extensively associated with lower cognitive performance in multiple epidemiological studies. Nevertheless, the exact impact of the presence of black carbon in the different brain regions and its effect(s) on neurobehavioral outcomes must be further elucidated in follow-up studies. Finally, we were able to confirm the carbonaceous nature of the identified black carbon particles through validation experiments (ie, emission signals stretched in the visible spectrum with an instantaneous response) and therefore exclude external contamination.

In contradistinction to the outcomes reported by Min et al,^17^ wherein the absence of black carbon particles was noted across diverse brain regions, with only marginal quantities detected in an olfactory nerve sample, our investigation demonstrated the detection of black carbon particles within all the included human brain regions. In contrast to the mentioned laser desorption/ionization mass spectrometry (LDI-MS) imaging method, our already proven white light technique offers a distinct advantage in terms of its ability to count individual particles, thereby providing a more direct and precise means of assessing black carbon presence.^10,12,13^ While LDI-MS is a powerful tool for the identification of chemical components, it may not capture the spatial distribution and concentration of individual particles, particularly at lower concentrations.

### Limitations

While the findings of this study enhance our understanding of potential adverse pathways associated with the suggested correlation between air pollution exposure and brain health, it is imperative to interpret these results within the context of the study’s limitations, including the modest sample size that necessitates cautious interpretation within an exploratory framework. The exploratory nature implies a preliminary exploration of black carbon accumulation in the human brain, and the absence of residential exposure data limits a comprehensive analysis. Nevertheless, these findings offer valuable insights into the translocation of soot exposure to critical brain areas, encouraging further exploration into the direct impact of air pollution, particularly combustion-derived particles, on human brain development and aging. Future research endeavors encompassing larger cohorts and including brains affected by various stages of neurodegeneration across diverse populations will be instrumental in refining and expanding upon the insights gleaned from this preliminary exploration.

## Conclusions

In this case series, we not only showed the presence of black carbon particles in the brain, but we also detected a significantly higher number of black carbon particles in memory-related brain regions like the thalamus, the prefrontal cortex including the olfactory bulb, and the hippocampus compared with the cingulate cortex, amygdala, and the superior temporal gyrus. Our study provides compelling evidence to further support the link between air pollution and the potential negative effects of ambient air pollutants on central nervous system disorders. Nevertheless, further research is needed on a bigger sample size in combination with the inclusion of healthy controls to demonstrate a causal relationship between the presence of black carbon in the brain and the onset and progression of Alzheimer disease.

## Notes

Supplement.
Data Sharing Statement
