# Supplementary material for: Accumulation of Ambient Black Carbon Particles Within Key Memory-Related Brain Regions
Source: JAMA Netw Open. 2024 Apr 9;7(4):e245678. doi: 10.1001/jamanetworkopen.2024.5678 (PMC11004827; doi:10.1001/jamanetworkopen.2024.5678)
Supplement: Supplement. — Data Sharing Statement [file jamanetwopen-e245678-s001.pdf]

Data Sharing Statement

Vanbrabant. Accumulation of Ambient Black Carbon Particles Within Key Memory-Related Brain Regions. *JAMA Netw Open*. Published April 09, 2024.
doi:10.1001/jamanetworkopen.2024.5678

Data
Data available: Yes
Data types: Data (not involving human participants)
How to access data: Data will be made available upon reasonable request to the corresponding author (kenneth.vanbrabant@uhasselt.be).
When available: With publication

Supporting Documents
Document types: None

Additional Information
Who can access the data: anyone upon reasonable request
Types of analyses: For scientific purposes
Mechanisms of data availability: after approval of a proposal
